# Reading and language intervention for children at risk of dyslexia: a randomised controlled trial

**DOI:** 10.1111/jcpp.12257

**Published:** 2014-05-17

**Authors:** Fiona J Duff, Charles Hulme, Katy Grainger, Samantha J Hardwick, Jeremy NV Miles, Margaret J Snowling

**Affiliations:** 1 Department of Experimental Psychology, University of OxfordOxford, UK; 2 Division of Psychology and Language Science, University College LondonLondon, UK; 3 Department of Psychology, University of YorkYork, UK; 4 RAND CorporationSanta Monica, CA, USA; 5 St. John's College, University of OxfordOxford, UK

**Keywords:** Dyslexia, specific language impairment, reading, intervention, RCT design

## Abstract

**Background:**

Intervention studies for children at risk of dyslexia have typically been delivered preschool, and show short-term effects on letter knowledge and phoneme awareness, with little transfer to literacy.

**Methods:**

This randomised controlled trial evaluated the effectiveness of a reading and language intervention for 6-year-old children identified by research criteria as being at risk of dyslexia (*n* = 56), and their school-identified peers (*n* = 89). An Experimental group received two 9-week blocks of daily intervention delivered by trained teaching assistants; the Control group received 9 weeks of typical classroom instruction, followed by 9 weeks of intervention.

**Results:**

Following mixed effects regression models and path analyses, small-to-moderate effects were shown on letter knowledge, phoneme awareness and taught vocabulary. However, these were fragile and short lived, and there was no reliable effect on the primary outcome of word-level reading.

**Conclusions:**

This new intervention was theoretically motivated and based on previous successful interventions, yet failed to show reliable effects on language and literacy measures following a rigorous evaluation. We suggest that the intervention may have been too short to yield improvements in oral language; and that literacy instruction in and beyond the classroom may have weakened training effects. We argue that reporting of null results makes an important contribution in terms of raising standards both of trial reporting and educational practice.

## Introduction

Dyslexia is a heritable learning disorder that primarily affects accurate and fluent word reading and spelling (Peterson & Pennington, 2012). It is also a common developmental outcome of preschool language impairment (Catts, Fey, Zhang, & Tomblin, 1999; Snowling, Bishop, & Stothard, 2000). Considerable research has addressed how to improve the literacy skills of children with dyslexic difficulties (National Reading Panel, 2000; Torgerson, Brooks, & Hall, 2006). The evidence shows that effective reading interventions incorporate training in letter–sound knowledge and phoneme awareness, explicit and systematic phonics instruction, and the application of these skills to the tasks of reading and spelling. This intervention approach is consistent with a theoretical model which views letter–sound knowledge and phoneme awareness as two causal influences on learning to read (Hulme, Bowyer-Crane, Carroll, Duff, & Snowling, 2012).

Few intervention studies have specifically targeted children who are at family risk (FR) of dyslexia (for summary, see van Otterloo & van der Leij, 2009). In the existing studies (conducted in English, Danish and Dutch), the interventions were implemented prior to the onset of formal reading instruction and focused on training the two key foundations for learning to read – letter knowledge and phoneme awareness – with some programmes including links to basic decoding and encoding processes. Several of the studies suffer methodological or statistical flaws – notably lack of random assignment of children to groups, or failure to control baseline skills in longitudinal analyses. Nonetheless, some general conclusions can be drawn.

Intervention efficacy is typically tested by comparing the progress of FR children who receive intervention with that of FR children who do not (forming either a treated or untreated control group). At immediate posttest, the intervention groups usually show a significant advantage in letter knowledge and phoneme awareness (Elbro & Petersen, 2004; Hindson et al., 2005; van Otterloo & van der Leij, 2009; van Otterloo, van der Leij, & Henrichs, 2009; Regtvoort & van der Leij, 2007). In some cases, progress on these measures is enough to bring FR children in line with their classmates who are deemed not to be at risk of reading difficulties (Elbro & Petersen, 2004; Hindson et al., 2005; Regtvoort & van der Leij, 2007). However, such training confers little measurable advantage for literacy development, as shown by the lack of significant differences between trained and untrained FR children on measures of reading and spelling at follow-up (typically 6–24 months later). A corollary of this is that the literacy skills of FR children – regardless of training – tend to fall significantly behind those of their unaffected peers (Hindson et al., 2005; Regtvoort & van der Leij, 2007).

An exception to these findings comes from Elbro and Petersen (2004), who evaluated the effect of daily training in phoneme awareness linked to letter–sound knowledge, delivered for 17 weeks during Kindergarten. In Grades 2 and 3, trained FR children had made more progress than untrained FR children in word and nonword reading accuracy and efficiency (controlling for baseline phoneme awareness and letter knowledge). The advantage on efficiency measures remained in Grade 7. Furthermore, in Grade 2, there were no significant differences in reading skills between trained FR children and their untrained, unaffected peers. Although the gap between those with and without FR widened over time, the two groups remained comparable on measures of word reading efficiency and reading comprehension in Grade 7.

In summary, interventions for children at family risk of dyslexia that are delivered before the onset of formal reading instruction tend to show short-term effects on phoneme awareness and letter knowledge. Though there are exceptions, these initial benefits seem not to transfer to higher level literacy skills.

Here, we report a randomised controlled trial (RCT) of a combined reading and language intervention, delivered alongside formal literacy instruction to 6-year-old children at risk of developing dyslexia. Consistent with the known risk factors for dyslexia, the research criteria for being at risk were as follows: having a first degree relative with dyslexia, and/or the presence of a preschool language impairment (see Nash, Hulme, Gooch, & Snowling, 2013). Children identified by schools as being in need of additional reading support also participated. Little work has been done to systematically examine the profiles of children at risk of reading difficulty for different reasons. However, findings suggest that school-age poor readers show similar patterns of language and literacy impairments regardless of whether they have a family risk for dyslexia (Carroll, Mundy, & Cunningham, 2014). Analyses of prereading language profiles suggest that children with a language impairment (regardless of family risk for dyslexia) are more likely to develop a reading difficulty than children with family risk alone (Nash et al., 2013).

Given the nature of our sample, we wished to address children's language and literacy difficulties. We based the intervention on our previous research demonstrating that reading and its foundations can be improved through phonological approaches (e.g. Bowyer-Crane et al., 2008; Hatcher et al., 2006), and that interventions incorporating training in vocabulary and oral narrative skills are effective for promoting oral language skills (e.g. Bowyer-Crane et al., 2008; Fricke, Bowyer-Crane, Haley, Hulme, & Snowling, 2013). We employed a waiting list control design and hypothesised that children receiving the intervention (Experimental group) would make significantly greater progress in reading and language skills than children not initially receiving the intervention (Waiting Control group). We expected that once the Waiting Control group received intervention, its progress would be comparable to that seen in the Experimental group – acting as a replication. We also expected to observe a dosage effect – with greater gains made following 18 weeks versus 9 weeks of intervention.

## Method

This study formed part of a longitudinal at-risk project investigating the development of dyslexia and language impairment (Wellcome Language and Reading Project). Children were screened in their first or second year of school to identify those with the weakest reading skills. Selected children took part in this RCT of a reading and language intervention. They were randomly allocated to the Experimental group (18 weeks of intervention) or Waiting Control group (standard classroom education for 9 weeks, followed by 9 weeks of intervention). Children were assessed individually at initial screening (*t0*), pretest (*t1*), midtest (*t2*, after 9 weeks of intervention for the Experimental group and 0 weeks for the Control group) and posttest (*t3*, after 18 or 9 weeks of intervention respectively).

Ethical permission was granted by the NHS Research Ethics Committee and the Psychology Department, University of York. Informed written consent was obtained from children's parents and from head teachers.

### Participants

Children were recruited to the longitudinal project on the basis of being at family risk of dyslexia and/or having preschool language impairment (see Nash et al., 2013 for details). Of 209 eligible children, 171 were available for screening (*t0*) in December 2010 to determine suitability for the intervention [mean age = 6.00 (0.06)]. Children were assessed on two tests of word reading from the *York Assessment of Reading for Comprehension* (*YARC*): *Early Word Reading* (*EWR*, Hulme et al., 2009) and *Single Word Reading* (*SWR*, Snowling et al., 2009). Mean standard scores were 104.08 (14.32) and 97.31 (16.50) respectively. The children were ranked in order of their average standard score, and 61 children with the lowest scores were selected.^1^ These children were randomly allocated, at the level of the child, to the Control (*n* = 30) or Experimental (*n* = 31) groups. The frequency of different types of risk (family risk (FR) vs. language impairment (LI) [or speech sound disorder (SSD)]) was similar across groups (*χ*^*2*^ = 0.92, *p *=* *.820). Comparing the Control and Experimental groups, respectively, rates of classification were: 13 versus 11 for FR; 7 versus 8 for LI; 5 versus 6 for FR + LI; 2 versus 4 for SSD.

The selected children were distributed across 44 schools: 37 schools with one child, six schools with two and one school with seven. For each child that the research team identified for intervention, their school was invited to nominate two additional children. The only inclusion criteria were that these children should be deemed likely to benefit from a reading intervention, and able to work well with the identified child. These additional children were assigned to the same group as that of the child in their school whom the research team had identified. There was variation in uptake, resulting in 51 additional children in the Experimental group and 46 in the Control group.

**Figure 1:**
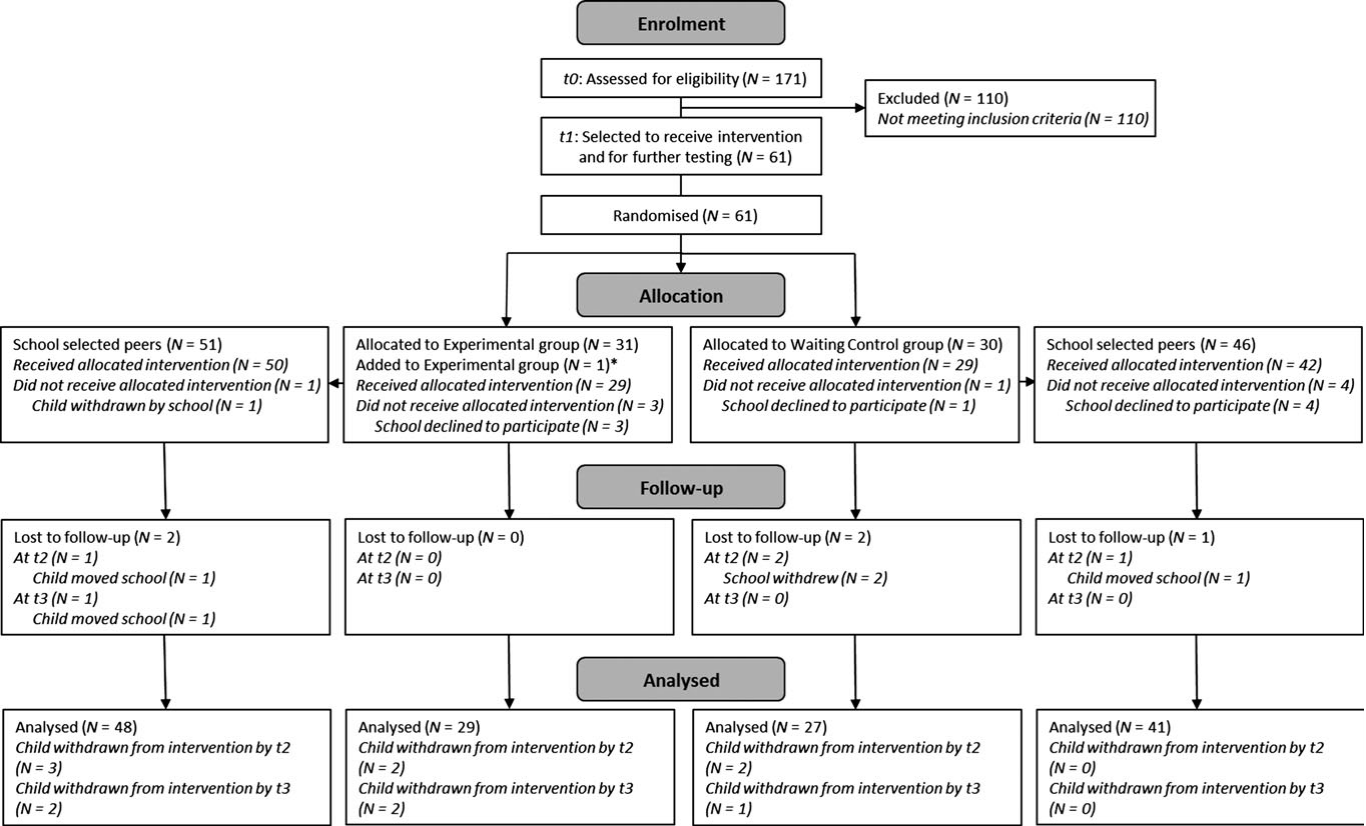
CONSORT diagram showing the flow of participants through the trial. *Note that after randomisation, one of the school selected peers was in fact involved in the longitudinal research project

Figure1 details the flow of participants through the trial, in accordance with CONSORT guidelines (Schulz, Altman, & Moher, 2010). Complete data sets were collected for 77 children in the Experimental group (42 boys; 29 children from the original at-risk sample) and 68 in the Control group (45 boys; 27 from the at-risk sample). Of these, 68 and 67, respectively, remained in the intervention for the prescribed length. Reasons for withdrawing from the intervention included children leaving schools, teaching assistants (TAs) leaving schools or taking sick leave, or school personnel deciding to withdraw children. However, all children with complete data sets were included in the analyses regardless of the extent to which they received the intervention. Preintervention (*t1*), the two groups (Control vs. Experimental) were of a similar age [6.04 (0.06) vs. 6.06 (0.07)]. Their standard reading and vocabulary scores were similar and in the low-average range: *EWR* – 91.75 (10.52) versus 91.22 (11.78); *SWR* – 85.51 (13.79) versus 84.36 (13.80); *Expressive Vocabulary* – 93.74 (17.45) versus 92.75 (20.12).

### Measures

Brief details of the test battery are given below (See Appendix S1, for full details).

The following standardised tests were administered at *t1, t2* and *t3*, as per the testing manuals: *Letter*–*Sound Knowledge, Sound Deletion, Early Word Reading* (also at *t0*)*, Single Word Reading* (also at *t0*) and *Passage Reading* (yielding measures of *Prose Reading Accuracy* and *Reading Comprehension*) from the *YARC* (Hulme et al., 2009; Snowling et al., 2009); the *Graded Nonword Reading Test* (Snowling, Stothard, & McLean, 1996); and *Expressive Vocabulary* (picture naming) from the *Clinical Evaluation of Language Fundamentals IV* (Semel, Wiig, & Secord, 2003).

The following tasks were also administered at *t1, t2* and *t3*.

#### Phoneme awareness

The phoneme blending and segmentation subscales from the *Sound Linkage Test of Phonological Awareness* (Hatcher, 2000) were administered by TAs.

#### Spelling

Children provided spellings of 10 pictured items (after Hulme et al., 2012). An *Orthographic Spelling* score was computed based on whole-word representations, and a *Phonetic Spelling* score based on how closely children's orthographic representations of the consonants matched the target consonants.

#### Taught vocabulary

To assess knowledge of words taught in the intervention, children gave definitions of 24 words (12 targeted in weeks 1–9 of intervention, 12 in weeks 10–18).

#### Listening comprehension

Children listened to and answered questions about two short stories.

### Intervention programme

A new intervention programme – *Reading and Language Intervention* (RALI) – was devised by the research team and implemented by TAs who already worked in the participating schools. The daily sessions alternated between 20-min individual sessions (Reading Strand) and 30-min small group sessions (Language Strand), with three individual and two group sessions per week. Groups included two to four children, with a mode of 3. All sessions followed a standard procedure, within which content was tailored to individual children's needs.

The Reading Strand was an abbreviated version of *Reading Intervention* (Hatcher et al., 2006), an evidence-based programme which integrates training in phonological awareness and reading. The Language Strand focused on training vocabulary and narrative skills and was adapted from previous interventions (e.g. Bowyer-Crane et al., 2008; Fricke et al., 2013), but used storybooks as the foundation for its themes and structure. An outline of the structure and contents of the sessions is given in Table1 (see Appendix S2 for more details).

**Table 1:** Content of group and individual sessions in reading and language intervention

Group sessions (A, B, C) – Language Strand (30 min)		Individual session – Reading strand (20 min)	
Active Listening (A)/Revision (B, C) 7 min (Session A) 2 min (Session B & C)	Storybook reading and introduction (A). Recap of story content and target words (B). Recap of story structure and story elements (C).	Easy Book Reading (2–3 min)	The child reads a familiar book which can be read with >94% accuracy. In all reading activities, phonic decoding is encouraged as the primary strategy for reading unknown words; other strategies (e.g. use of context and pictures) are also taught.
Vocabulary Instruction (A, B, C) 10 min (Session A) 15 min (Session B) 8 min (Session C)	Explicit, multicontextual and interactive teaching of two target words (A) or three target words (B) from the book. Explicit, multicontextual and interactive consolidation of the five target words (C).	Instructional Book Reading (5 min)	The child is assessed while reading a book at the instructional level (90–94% accuracy). Teaching points related to the child's reading strategies follow.
Spoken and Written Narrative (A, B, C) 12 min (Session A & B) 19 min (Session C)	Retelling the story and shared writing (A). Work on story elements; retelling the story and shared writing (B). Retelling the story, shared writing and guided/independent writing; prediction of story endings (C).	Sight Word Learning (2–3 min)	The child learns irregular and high frequency words through multisensory teaching methods.
Plenary (A, B, C) 1 min (Session A, B, C)	Recall of target words (A, B, C).	Letters, Sounds and Linkage (5 min)	The child is trained in letter knowledge (if necessary). Phonological awareness training focuses on manipulating phonemes. Phoneme awareness is linked to letters and words through phonic decoding and encoding exercises.
		New Book Reading (5 min)	The plot and characters of a new book at the instructional level are discussed. The teaching assistant scaffolds the child's first attempt at reading this new book.

Treatment fidelity was monitored in various ways. TAs attended 2.5 days of training by the research team prior to intervention delivery, and received a manual containing scripted lesson plans for the Language Strand and detailed guidance for the Reading Strand. TAs submitted attendance registers and lesson plans. They received email or telephone support from the research team throughout their involvement (fortnightly contact in the first 9 weeks of delivery; monthly support in the second 9 weeks). Finally, every TA was observed delivering at least one teaching session, and given constructive feedback. During observations, components were graded on a 3-point scale according to quality of teaching (1 = poor, 2 = satisfactory, 3 = good). The maximum teaching score was 15 for the Reading Strand; Experimental group TAs scored on average 10.80 (2.33) and Control group TAs 10.14 (2.32). The maximum teaching score for the Language Strand was 12; Experimental group TAs scored on average 9.47 (1.74) and Control group TAs 9.71 (1.70).

## Results

Table2 reports the raw scores for both groups on all outcome measures at *t1*, *t2* and *t3*. The effects of the intervention on language and literacy outcomes were assessed using regression (ANCOVA) models in Stata (Ver. 11; StataCorp, 2009). Bootstrapping (with 1,000 replications) was applied to all analyses to ensure accurate standard errors when residuals were nonnormally distributed (Efron & Tibshirani, 1993). Analyses were performed on the full sample, and the at-risk subsample. Mixed effects (multilevel) models were run (xtmixed) to account for clustering at the school level in the full sample. The assumption of homogeneity of regression slopes was tested by including the group × covariate interaction term in the models (retaining it when significant).

**Table 2:** Means (*SD*s) and ranges on all outcome measures at *t*1, *t*2 and *t*3 for the control and experimental groups

Outcome measure (maximum)	Reliability	Test point	Control		Experimental	
			Mean	Range	Mean	Range
Letter knowledge (32)	.98^a^	*t*1	28.59 (4.59)	8–32	27.53 (3.79)	14–32
		*t*2	29.68 (4.05)	6–32	30.09 (2.70)	17–32
		*t*3	30.71 (3.13)	11–32	30.68 (2.20)	19–32
Phoneme awareness (12)	.64–.78^b^	*t*1	8.91 (2.93)	0–12	7.97 (2.88)	0–12
		*t*2	9.38 (3.09)	0–12	10.30 (2.04)	2–12
		*t*3	10.73 (2.30)	1–12	10.97 (1.8)9	1–12
Sound deletion (12)	.93^a^	*t*1	5.63 (2.65)	0–12	5.74 (2.03)	1–10
		*t*2	6.84 (2.24)	2–11	6.57 (2.45)	0–11
		*t*3	7.60 (2.68)	0–12	7.55 (2.26)	1–12
Early word reading (30)	.98^a^	*t*1	16.13 (8.50)	0–29	16.70 (8.34)	1–30
		*t*2	19.41 (8.57)	0–30	20.97 (7.07)	4–30
		*t*3	23.09 (7.90)	0–30	23.74 (6.55)	1–30
Single word reading (60)	.98^a^	*t*1	9.43 (7.56)	0–28	9.60 (7.65)	0–32
		*t*2	13.35 (9.49)	0–36	13.96 (8.33)	0–33
		*t*3	18.38 (10.71)	0–40	18.62 (9.95)	0–40
Nonword reading (20)	.96^a^	*t*1	4.46 (4.89)	0–16	3.67 (4.01)	0–14
		*t*2	6.29 (5.66)	0–19	5.69 (4.57)	0–18
		*t*3	8.19 (5.70)	0–20	8.17 (5.49)	0–20
Prose reading accuracy (48)†	.75–.87^a^	*t*1	36.62 (10.27)	9–48	37.23 (9.27)	10–48
		*t*2	30.41 (13.94)	2–48	28.97 (12.77)	3–48
		*t*3	22.18 (15.10)	0–48	22.18 (14.34)	0–46
Orthographic spelling (10)	.66–.76	*t*1	2.62 (1.73)	0–7	3.14 (1.73)	0–10
		*t*2	3.47 (1.72)	0–8	3.74 (1.83)	0–9
		*t*3	4.40 (2.39)	0–10	4.55 (2.12)	0–10
Phonetic spelling (92)	.87–.92^b^	*t*1	72.24 (22.83)	0–92	78.21 (18.08)	0–92
	.99^c^	*t*2	80.34 (17.65)	0–92	82.96 (13.23)	0–92
		*t*3	81.79 (19.87)	0–92	86.08 (11.45)	0–92
Expressive vocabulary (54)	.85^a^	*t*1	25.66 (9.26)	2–46	26.56 (9.51)	2–47
		*t*2	29.12 (9.22)	2–44	29.31 (8.29)	10–48
		*t*3	31.29 (9.27)	2–50	31.05 (8.88)	6–50
Taught vocabulary weeks 1–9 (36)	.73–.84^b^	*t*1	13.28 (5.21)	0–26	13.21 (5.02)	0–23
		*t*2	14.87 (5.69)	0–27	17.06 (5.29)	2–29
		*t*3	18.34 (5.45)	0–27	17.26 (5.45)	2–28
Taught vocabulary weeks 10–18 (36)	.73–.84^b^	*t*1	14.88 (4.83)	0–25	14.12 (5.13)	0–25
	.83–.90^d^	*t*2	16.00 (5.17)	0–27	16.06 (4.74)	5–25
		*t*3	17.81 (5.23)	0–28	18.36 (5.53)	0–30
Listening comprehension (17)	.65–.71^b^	*t*1	7.15 (2.97)	0–14	7.22 (2.76)	2–13
		*t*2	8.71 (3.06)	0–14	8.60 (2.40)	4–14
		*t*3	9.00 (2.78)	1–15	9.16 (2.70)	2–14
Reading comprehension (24)	.62–.77^a^	*t*1	6.28 (5.50)	0–21	5.89 (4.46)	0–18
		*t*2	8.94 (6.22)	0–21	9.58 (5.90)	0–23
		*t*3	11.68 (6.14)	0–21	12.17 (5.99)	0–22
Sessions attended (45)		*t*1–*t*2	0.00 (0.00)	0–0	36.18 (7.64)	0–46
		*t*2–*t*3	39.10 (4.99)	24–46	33.01 (13.39)	0–47

† Lower scores denote fewer errors and therefore greater accuracy.

a Internal reliability (*α*).

b Test/retest reliability.

c Intrarater reliability.

d Interrater reliability.

**Figure 2:**
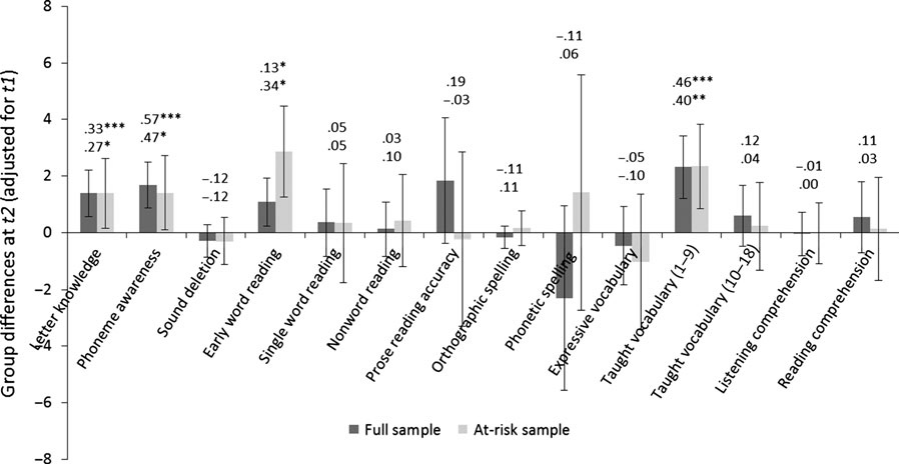
Relative advantage for the Experimental versus Control group in *t2* marginal mean scores on language and literacy outcomes (with 95% confidence intervals). Effect sizes are above the bars; uppermost values refer to the full sample, lowermost to the at-risk subsample. Note: The *y*-axis for letter knowledge and phonetic spelling represents instead the average difference in raw score gains across *t1* and *t2* between the groups

The effect of the intervention was first assessed by testing for group differences at *t2*, controlling for differences in baseline performance at *t1*.^2^ These analyses test whether the children receiving the intervention (Experimental group) progress more in the first 9-week period than the children not receiving the intervention (Control group). The results are summarised in Figure2, which plots the difference between the groups' marginal means at *t2* (controlling for *t1* covariates). A score greater than 0 signifies more progress in the Experimental group than the Control group; where the 95% confidence intervals do not cross the *x*-axis, this represents a statistically significant effect (*p *<* *.05). Effect sizes (*d*) are shown. Figure2 demonstrates that the pattern of effects is similar across the full sample and the subsample. There are statistically significant but small effects^3^ on letter knowledge and early word reading, and small-to-moderate effects on phoneme awareness and taught vocabulary. After correcting for multiple comparisons (Benjamini & Hochberg, 1995), all effects in the full sample remain significant, but the effects observed on letter knowledge and phoneme awareness in the subsample are not significant. However, there is no effect of intervention on the majority of language and literacy measures. It should be noted that across the full sample and subsample, we had high power to detect medium effects but low power to detect small effects. Regarding the full sample, with an average pretest/posttest correlation of .75 using ANCOVA, we had 99.5% power to detect a group difference where *d *=* *0.50 and 44.3% power where *d *=* *0.20 (two-tailed). For the subsample, the average pretest/posttest correlation was .78, and the corresponding power values were 84.8% and 44.3%.

**Figure 3:**
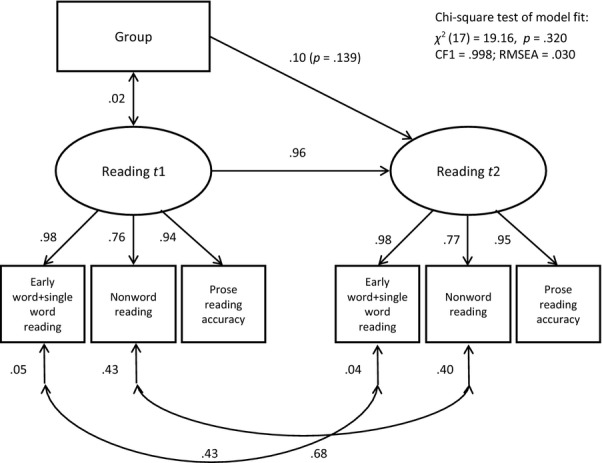
A path model for the full sample showing the effect of ‘group’ (proxy for intervention) on the latent variable ‘reading’ at *t2*, controlling for reading at *t1*

As children were initially selected for intervention because of risk of word-level reading difficulties, our primary outcome measures concerned word-level reading (early word reading, single word reading, nonword reading and prose reading accuracy). A significant effect of intervention was only detected on one of these four measures. To provide a robust test of the hypothesis that the intervention improved the primary outcome, we performed a path analysis with structural equation modelling to test the effect of group (proxy for intervention) on a latent ‘reading’ variable. The latent variable was formed from all four word-level reading measures: Strict factorial invariance was imposed; and to address the effects of nonnormal distribution, a composite was formed from the tests of early word reading and single word reading, and the prose reading accuracy measure was transformed by taking the square root (nonword reading was left unadjusted). The model concerns the full sample so accounted for clustering of children within schools. It provides a good fit to the data (see Figure3). The standardised coefficients demonstrate high stability in reading from *t1* to *t2*. For the paths from ‘group’ to ‘reading’, the partial regression coefficients (Y-standardised) are reported; these represent the difference in z-score units after controlling for the covariate and are equivalent to effect sizes. This model shows that after 9 weeks, there is no reliable effect of the intervention (‘group’) on word-level reading abilities (*d *=* *0.10).

**Figure 4:**
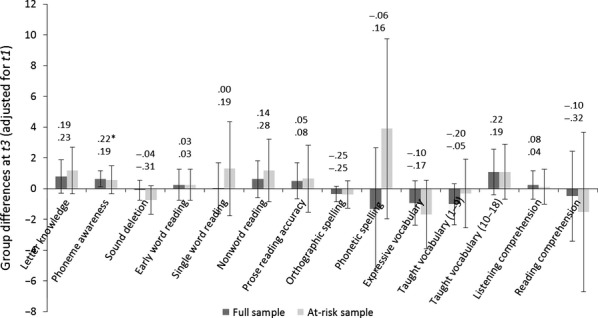
Relative advantage for the Experimental versus Control group in *t3* marginal mean scores on language and literacy outcomes (with 95% confidence intervals). Effect sizes are above the bars; uppermost values refer to the full sample, lowermost to the at-risk subsample. Note: The *y*-axis for letter knowledge and phonetic spelling represents instead the average difference in raw score gains across *t1* and *t3* between the groups

**Figure 5:**
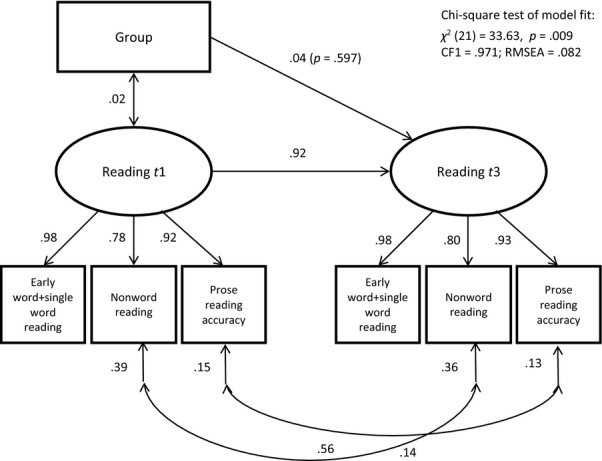
A path model for the full sample showing the effect of ‘group’ (proxy for intervention) on the latent variable ‘reading’ at *t3*, controlling for reading at *t1*

A second set of analyses was performed by testing for group differences at *t3,* controlling for differences in baseline performance at *t1*. These analyses test whether there is a benefit of receiving 18 versus 9 weeks of intervention (Experimental vs. Control group). The models were conducted as before (see Figure4). There is one statistically significant intervention effect – on phoneme awareness in the full sample – but this fails to survive the Benjamini–Hochberg correction. In short, there are no significant differences between the groups on any language or literacy measures at *t3*. Figure5 depicts the path analysis exploring the effect of ‘group’ on ‘reading’ at *t3*. Although a less good fit to the data has been achieved, the model makes clear that there is no reliable effect of intervention (‘group’) on word-level reading abilities at *t3* (*d *=* *0.04).

Finally, we tested whether the nature of children's risk for dyslexia affected response to intervention over the 18-week period by running regression models with bootstrapping (1,000 replications). Measures of growth were derived by computing residualised *t3* scores (controlling for *t1*) on a composite measure of word reading accuracy (early word reading and single word reading combined) and a composite measure of taught vocabulary (weeks 1–9 and weeks 10–18 combined). These growth measures were predicted by risk classification [using dummy codes for family risk only (0,1), preschool language impairment only (0,1), family risk and language impairment combined (0,1)]. Neither of the models accounted for significant variance in growth; and none of the variables was a unique predictor (see Table3), indicating that response to intervention was not determined by the nature of children's risk for dyslexia.

**Table 3:** Regression models predicting growth in reading and vocabulary over 18 weeks

Predictor	*β*	*SE*	*Z*	*p*
Model 1: Reading growth				
Family risk (FR)	1.11	1.71	0.65	.516
Language impairment (LI)	0.62	3.53	0.18	.860
FR + LI	1.16	4.16	0.28	.780
Model 2: Vocabulary growth				
FR	2.77	1.63	1.70	.090
LI	2.61	2.14	1.22	.221
FR + LI	−1.17	0.71	−1.65	.100

## Discussion

This randomised controlled trial evaluated a reading and language intervention delivered to 6-year-old children at risk of developing dyslexia, taught alongside classmates selected by teachers as likely to benefit from extra literacy support. The content of the intervention was chosen to reflect our novel approach to conceptualising risk; that is, with respect to family history, but also the presence of preschool language impairment. We found only small-to-moderate effects of the intervention on foundational skills that received explicit training (letter knowledge, phoneme awareness and taught vocabulary), but these effects were fragile and short lived, and there was no transfer to our primary outcome of word-level reading. The nature of dyslexia risk (family history and/or language impairment) did not affect response to intervention. The pattern of short-term effects on letter knowledge and phoneme awareness with no transfer to word-level literacy characterises the majority of family risk intervention studies. However, the effects of the intervention were similarly small and circumscribed across the broader sample that also included children identified by teachers as needing reading intervention (though we acknowledge the limitation of not knowing the criteria used to select these additional children). The intervention presented here was theoretically motivated and based on previous successful interventions (e.g. Bowyer-Crane et al., 2008; Hatcher et al., 2006). Thus, we are left with the question of why the intervention did not have reliable and consistent effects on language and literacy skills.

One possibility is that the 9-week period was simply too short. This seems a valid argument with respect to training broader oral language skills. Significant effects have been observed on a narrative composite and a broader oral language composite (untrained vocabulary, listening comprehension, and content and grammar of spoken language) after a 30-week intervention, but only on grammar and trained vocabulary (not untrained vocabulary, listening comprehension or narrative skill) after a 20-week intervention (Bowyer-Crane et al., 2008; Fricke et al., 2013). Furthermore, with respect to the Language Strand, although narrative work could be differentiated based on individual needs, vocabulary instruction had to be more uniform given the pretest/posttest.

The relatively short length of the intervention does not seem a sufficient explanation for the limited effects on word-level reading. The Reading Strand was based on the effective programme of Hatcher et al. (2006), in which the Experimental group gained 4.59 standard score points on a test of word reading in its first 10-week intervention period (0.28 points per hour of intervention) and the Control group 0.86 points (*d *=* *0.69). In the first 9 weeks of the present intervention, the Experimental group gained 4.13 standard score points on the *Single Word Reading Test* (0.23 points per hour). Taken alone, this comparison suggests the Experimental group here made sufficient progress. However, in the same 9 weeks, the Control group gained 3.49 standard score points in the absence of intervention, resulting in a weak intervention effect (*d *=* *0.10).

This pattern suggests that the Control group might not have provided a pure comparison. Indeed, post hoc analysis of teacher questionnaire data showed that the majority of the full sample (>90%) was receiving systematic phonics instruction as part of their classroom literacy instruction. A weakened intervention effect against this instructional backdrop might therefore be a positive reflection of policy aims to raise the quality of baseline classroom instruction for all (e.g. Rose, 2006). It is possible that the Reading Strand of the intervention might not have differed sufficiently from the classroom literacy approach to induce larger and more sustained intervention effects. Furthermore, the at-risk subsample in this study had been part of a longitudinal project since the age of 3, and therefore comprised children with engaged and informed parents, many of whom provided literacy support at home. In addition, across the full sample, teacher questionnaire data revealed that 51% of the Experimental group and 54% of the Control group were already receiving some form of additional literacy support when the current intervention began (with modal responses characterising this as entailing approximately 20 min of TA-delivered literacy instruction one or five times a week). Finally, while a phonics-based reading intervention at the age of 6 is a defensible strategy for young struggling readers, our heterogeneous sample included some children whose initial reading abilities were already beyond the level that would be targeted in the intervention – possibly constraining the growth they could demonstrate on the outcome measures. Given the methodological difficulties of carrying out this trial with sufficient power, these sample characteristics were regrettable but unavoidable.

While we can offer various conjectures, our only definitive conclusion is that the intervention, as delivered under the circumstances reported here, did not produce reliable improvements in children's language and literacy skills. However, this does not render reporting of the trial unimportant. Our findings highlight the importance of subjecting well-intended educational practices to rigorous evaluation; and emphasise the need for practitioners to understand and implement evidence-based approaches (Duff & Clarke, 2011). Such objectives accord with the drive to bring standards for reporting nonpharmacological trials in line with pharmacological trials (Science & Technology Committee, 2013).

## Conclusion

We have reported an RCT of an intervention for 6-year-old children identified by research criteria as being at risk of dyslexia, and their school-identified peers. The intervention was theoretically motivated and based on previous successful interventions. We found evidence for small-to-moderate effects after 9 weeks of intervention for the Experimental group on letter knowledge, phoneme awareness and vocabulary. However, these effects dissipated once the Control group had also received intervention. Critically, there was no significant effect on a robust latent measure of word-level reading. We have offered tentative explanations for this null result: The intervention may have been too short to expect significant improvements in language skills; and the nature and frequency of literacy instruction in and beyond the classroom may have weakened the effects on reading. Following previous at-risk intervention studies, small improvements were seen in letter knowledge and phoneme awareness, but with no transfer to literacy. Although disappointing, we believe reporting a null result is important with respect to raising standards in reporting of trials and in educational practice.

**Key Points**

- Previous interventions for children at family risk of dyslexia typically show short-term effects on letter knowledge and phoneme awareness but little transfer to literacy.
- That pattern was replicated following a critical 9-week period of a new reading and language intervention, but was not particular to children identified by research criteria as being at-risk of dyslexia.
- The intervention was not effective as delivered under the conditions reported here: It may have been too short to yield improvements in oral language; and literacy instruction in and beyond the classroom may have weakened training effects.
- Reporting of null results in non-pharmacological trials is important with respect to raising standards in trial reporting and educational practice.

## Note

1 Owing to an error, 61 children were selected instead of 60.

2 As letter–sound knowledge and phonetic spelling were severely affected by ceiling effects, differences in simple gain scores over time between the groups were analysed, rather than estimating regression slopes across a restricted range of values.

3 Effect sizes of 0.2 are considered small, 0.5 moderate and 0.8 large (Cohen, 1992).

## Supporting information

**Appendix S1.**Test Battery. **Appendix S2.**Intervention Programme.
