# Supplementary material for: Reading and language intervention for children at risk of dyslexia: a randomised controlled trial
Source: J Child Psychol Psychiatry. 2014 May 17;55(11):1234–43. doi: 10.1111/jcpp.12257 (PMC4368377; doi:10.1111/jcpp.12257)
Supplement: Supplementary file 1 — Appendix S1.Test Battery. Appendix S2.Intervention Programme. [file jcpp0055-1234-sd1.docx]

*Online supplementary file for:* ***Reading and Language Intervention for Children At-Risk of Dyslexia: A Randomised Controlled Trial****; by Duff et al*

[**Appendix S1. Test Battery**](#AppendixS1)

[**Appendix S2. Intervention Programme**](#AppendixS2)

[**References**](#refernces)

**Appendix S1. Test Battery**

***Measures***

*Letter-sound knowledge (t1, t2, t3).* This was assessed using the extended *Letter-sound Knowledge* sub-test from the *YARC* (Hulme et al., 2009). Children were asked to produce the sounds associated with the 26 letters of the alphabet, and 6 digraphs. Children were prompted for letter-sounds if letter names were first given; and only short vowels were accepted as correct (internal reliability, *α* = .98).

*Phoneme awareness (t1, t2, t3).* Two subscales (each with 6 items) from the *Sound Linkage Test of Phonological Awareness* (Hatcher, 2000) were used to measure ability to blend and segment phonemes (test/re-test reliability on present sample, *r* = .64 to .78). Tests were administered by teaching assistants (TAs).

*Sound deletion (t1, t2, t3).* As a further test of phonological awareness, the *YARC Sound Deletion* test (Hulme et al., 2009) was administered. This involves deleting sounds (syllables then phonemes) from 12 words; for example, children were asked to say ‘starfish’ and then to say it again without the ‘fish’ (internal reliability, *α* = .93).

*Early word reading (t0, t1, t2, t3).* Children were administered the *Early Word Reading* sub-test from the *YARC* (Hulme et al., 2009). This assessed children’s ability to read 15 regular and 15 irregular words that are frequently encountered during the early stages of learning to read. Testing was discontinued after 10 consecutive errors (internal reliability, *α* = .98).

*Single word reading (t0, t1, t2, t3).* The *Single Word Reading* test (*SWR*) from the *YARC* (Snowling et al., 2009) was also administered. This comprises 60 words of increasing difficulty. Testing was discontinued after five consecutive errors (internal reliability, *α* = .98).

*Nonword reading (t1, t2, t3).* The *Graded Nonword Reading Test* (*GNWRT*; Snowling, Stothard, & McLean, 1996) was given as a measure of phonological decoding. The 20 items range from 4-8 graphemes in length. Testing was discontinued after six consecutive errors (internal reliability, *α* = .96).

*Spelling (t1, t2, t3).* Children were asked to name pictures of 10 items and to write down each associated word (after Hulme et al., 2012). An *Orthographic Spelling* score was computed – with 1 point awarded for each word spelt correctly (test/re-test reliability on present sample, *r* = .66 – .76). A *Phonetic Spelling* score was also derived, to assess how closely the orthographic representations of the consonants matched the target consonants: 4 points were awarded for each correct letter; 3 points for a letter that differed from the target consonant by only one phonetic feature (place, manner or voicing); 2 points for a partially represented digraph, or for a correct letter in the wrong position; and 1 point for a letter more than one phonetic feature away from the target consonant. Twenty-three consonants were scored, giving a total of 92 points (test/re-test reliability on present sample, *r* = .87 to .92; intra-rater reliability, *r* = .99).

*Taught vocabulary (t1, t2, t3).* Knowledge of words taught directly in the intervention was measured using a bespoke vocabulary test. Children were asked to give definitions for 24 words, half of which were targeted in weeks 1-9 of intervention and half in weeks 10-18. The test was delivered in 2 parts; each part containing 6 words taught in weeks 1-9 and 6 words taught in weeks 10-18. Words included nouns (e.g., competition, creature), verbs (e.g., collect, discover) and adjectives/adverbs (e.g., between, carefully). Responses were scored on a scale of 0 to 3 – according to the quality of definitions – giving a maximum score of 36 for each sub-test of words taught in weeks 1-9 and in weeks 10-19 (test/re-test reliability on present sample, *r* = .73 to .84; inter-rater reliability, *r* = .83 – .90).

*Expressive vocabulary (t1, t2, t3).* The standardised *Expressive Vocabulary* sub-test from the *Clinical Evaluation of Language Fundamentals IV* (*CELF IV* – Semel, Wiig, & Secord, 2003) was used to assess transfer of vocabulary skills. Children were asked to name a set of pictures which increased in difficulty (27 items each worth 2 points); testing was discontinued after seven consecutive errors (internal reliability, *α* = .85).

*Listening comprehension (t1, t2, t3).* This was assessed using the *York Assessment for Listening Comprehension* (*YALC* – Stothard, Snowling, & Hulme, unpublished research). Children listened to two short stories and after each were asked a set of comprehension questions, totalling 17 items (test/re-test reliability on present sample, *r* = .65 to .71).

*Reading Comprehension (t1, t2, t3).* The *YARC* *Passage Reading* test (Snowling at al., 2009) was used to measure reading comprehension. Children were asked to read three passages of increasing difficulty, and answered eight comprehension questions after each passage (internal reliability, *α* = .62 to .77). Testing was discontinued after 16 reading errors were made within a single passage; a measure of *Prose Reading Accuracy* was also derived (performance on parallel forms, *r* = .75 to .87). Passages not read due to discontinuation were credited with 16 reading errors and 0 points for comprehension.

**Appendix S2. Intervention Programme**

As children were selected for this intervention on the basis of having a risk for a reading difficulty owing to a family history of dyslexia and/or a pre-school language impairment, we devised a new intervention programme that incorporated training in both reading and language skills – the *Reading and Language Intervention* (RALI). The daily sessions alternated between 20-minute individual sessions (Reading Strand) and 30-minute small group sessions (Language Strand), with 3 individual and 2 group sessions per week. Individual sessions were chosen for the Reading Strand to ensure appropriate differentiation, while group sessions were chosen for the Language Strand in order to provide more opportunity for rich and natural language interactions. Including both code-focused (Reading Strand) and language-focused work (Language Strand) meant that children received training in the skills foundational to both reading accuracy and reading comprehension.

The Reading Strand was an abbreviated version of *Reading Intervention* (after Hatcher et al., 2006), an evidence-based programme which emphasises the importance of integrating training in phonological awareness with practising reading. In RALI, the individual sessions began with the child reading an ‘easy’ level book – that is, a book that can be read with above 94% accuracy.  The child’s ability to read an ‘instructional’ level book (90-94% accuracy) was then formally assessed, followed by the opportunity to make teaching points regarding reading strategies. Next, sight words were introduced and reinforced through multi-sensory activities. The following section focused on phonic skills. If letter knowledge was weak, letter-sounds were taught using multi-sensory techniques. Phonological awareness was trained, with a focus on manipulating phonemes through blending, segmentation, deletion and transposition activities. This phonemic knowledge was then explicitly linked to letters and words through phonic decoding and encoding work. Finally, the child was introduced to a new book at the ‘instructional’ level, with the Teaching Assistant (TA) scaffolding their first reading attempt. Throughout all reading activities, children were primarily encouraged to use phonic decoding skills when they encountered unknown words; however, other strategies such as the use of context were also taught.

The Language Strand was adapted from previous interventions (e.g. Bowyer-Crane et al., 2008; Fricke et al., 2013). In RALI, Storybooks provided the foundation for the themes and structure. This approach aimed to integrate the reading and language aspects of the intervention, and builds on other work in this area (e.g., Beck & McKeown, 2007; Coyne et al., 2009). For each 9 weeks of intervention, there were six associated storybooks, with two storybooks per theme. Themes related to the UK curriculum (Key Stage 1/2 QCA Schemes of Work) were selected (e.g. Outdoors, Growing, Feelings). Each storybook was the focus of teaching for three consecutive group sessions (Sessions A, B and C). All sessions targeted vocabulary and narrative skills, but the format of each session was slightly different.

The storybook was first introduced and read to the children at the beginning of Session A, and was used as a springboard for the rest of the teaching content. Each session included vocabulary instruction. Target words from the storybooks (including nouns, verbs, adjectives, prepositions) were taught using multi-contextual, multi-sensory approaches (e.g., Beck et al., 2002). New words were introduced in Sessions A and B, using a standard sequence: 1) placing the word in the context of the story; 2) asking children to guess the meaning of the word; 3) providing an age-appropriate definition, paired with a picture on a flashcard; 4) asking children to make judgements about the meaning of the word; 5) asking each child to use the word in a new sentence; 6) hearing and using the words in a vocabulary game; 7) recalling the words at the end of the current session and the beginning of the next session. In Session C, all target words from one storybook were revised through creating a word web: children were asked to recall the words, define them, describe them, and draw links between them. Narrative work also featured in each session, and was devised with reference to curriculum guidelines (Department for Education and Employment, 2001; Department for Education and Skills, 2006). In Session A, the children worked together to sequence three or four picture cards in the correct order from the storybook. In each session, the TA supported the group as they decided on a summary sentence to describe the picture representing the beginning (Session A), middle (Session B) and end (Session C) of the story. In a shared writing task, the TA asked the children to help transfer the sentence from speech to writing (emphasising phonic encoding). In Session C, this task progressed to independent writing: The TA set each child a separate writing task relating to the story sentences, which could range from writing single words to whole sentences. In Session B, work on story elements (character, setting, plot etc.) was also included.

**References**

Beck, I.L. & McKeown, M.G. (2007). Increasing young low-income children’s oral vocabulary repertoires through rich and focused intervention. *The Elementary School Journal, 107,* 251-271.

Beck, I.L., McKeown, M.G., & Kucan, L. (2002). *Bringing Words to Life: Robust Vocabulary Instruction.* The Guilford Press: New York.

Bowyer-Crane, C., Snowling, M.J., Duff, F.J., Fieldsend, E., Carroll, J.M., Miles, J., Götz, K., & Hulme, C. (2008). Improving early language and literacy skills: Differential effects of an oral language versus a phonology with reading intervention. Journal of Child Psychology & Psychiatry, 49, 422-432. DOI: 10.1111/j.1469-7610.2007.01849.x

Department for Education and Employment (2001). *Developing Early Writing.* London: DfEE Publications.

Department for Education and Skills (2006). *Primary Framework for Literacy and Mathematics.* London: DfES Publications.

Fricke, S., Bowyer-Crane, C., Haley, A., Hulme, C., & Snowling, M.J. (2013). Efficacy of language intervention in the early years. *Journal of Child Psychology and Psychiatry, 54,* 280-290. DOI: 10.1111/jcpp.12010

Hatcher, P.J., Hulme, C., Miles, J.N.V., Carroll, J.M., Hatcher, J., Gibbs, S., Smith, G., Bowyer-Crane, C., & Snowling, M.J. (2006). Efficacy of small-group reading intervention for beginning readers with reading delay: A randomised control trial. *Journal of Child Psychology and Psychiatry, 47*, 820-827. DOI: 10.1111/j.1469-7610.2005.01559.x

Coyne, M.D., McCoach, D.B., Loftus, S., Zipoli, R., & Kapp, S. (2009). Direct vocabulary instruction in kindergarten: Teaching for breadth versus depth. *The Elementary School Journal, 110,* 1-18.
